# Decoy fitness peaks, tumor suppression, and aging

**DOI:** 10.1111/acel.12938

**Published:** 2019-03-08

**Authors:** Kelly C. Higa, James DeGregori

**Affiliations:** ^1^ Department of Biochemistry and Molecular Genetics University of Colorado School of Medicine Aurora Colorado; ^2^ Integrated Department of Immunology University of Colorado School of Medicine Aurora Colorado; ^3^ Department of Pediatrics University of Colorado School of Medicine Aurora Colorado; ^4^ Department of Medicine, Section of Hematology University of Colorado School of Medicine Aurora Colorado

## Abstract

Recent reports by Martincorena et al and Yokoyama et al reveal unanticipated dynamics of somatic evolution in the esophageal epithelium, with clonal expansions apparently driven by mutations in Notch1 dominating the epithelium even in middle‐aged individuals, far outpacing the prevalence of these mutations in esophageal cancers. We propose a model whereby the promotion of clonal expansions by mutations such as in Notch1 can limit more malignant somatic evolutionary trajectories until old ages.

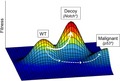

The recent papers by Martincorena et al and Yokoyama et al provide important and surprising new insights into how somatic cells in our tissues evolve with age (Martincorena et al., 2018; Yokoyama et al., 2019). These reports demonstrate strong selection for Notch1 mutations (likely loss of function) starting in middle age, with similar selection for TP53 gene disruption with a peak at older ages. While cancer risk factors like smoking and alcohol consumption exhibited a modest trend toward increased size of most mutational clonal expansions (such as involving Notch1 and PPM1D), clones with TP53 mutations were highly expanded in individuals with these risk factors (Yokoyama et al., 2019). Strikingly, while Notch1 mutant clones represent about half of the epithelial cells in individuals past 50 (Martincorena et al., 2018; Yokoyama et al., 2019), they are present in only ~10% of esophageal cancers. Multiple other genes exhibited greater mutational prevalence in normal esophageal epithelia than in esophageal carcinomas. Thus, it appears that a cancer is substantially more likely to initiate in a clone *without* a Notch1 mutation (as well as for other mutations, but we will use Notch1 mutation as the prototype here). So how could loss of Notch1 function impede cancer development?

Extrapolating from these results, we propose that Notch1 mutations are not actually “tumor suppressive” from a cell autonomous standpoint. Instead, we hypothesize that local adaptive peaks (“decoy fitness peaks”; such as occupied by clones with Notch1 mutations) can impede access to more malignant evolutionary trajectories, even if such decoy peaks can still lead, albeit with reduced frequency or delayed kinetics, to malignancy (Figure 1). The Notch1 mutant decoy peak could simply be less advanced on the path to cancer or may simply be less likely to evolve toward cancer. Occupancy of the decoy peak can reduce progression up the more malignant peak by rendering some oncogenic changes now disadvantageous or by reducing opportunities for further mutational adaptation. To put this simply, fit cells provide better competition for malignant cells. By providing adaptation to the age‐altered tissue landscape, Notch1 mutations improve the fitness of esophageal epithelial progenitors, filling up the niche with cells that are less likely to be further improved by additional cancer‐promoting mutations. In fact, previous studies demonstrated that Notch1 inactivation provides a competitive advantage in the mouse esophageal epithelium by decreasing differentiation, as well as by actively promoting differentiation of neighboring wild‐type cells (Alcolea et al., 2014), reminiscent of super‐competition in *Drosophila* (Merino, Levayer, & Moreno, 2016).

**Figure 1:**
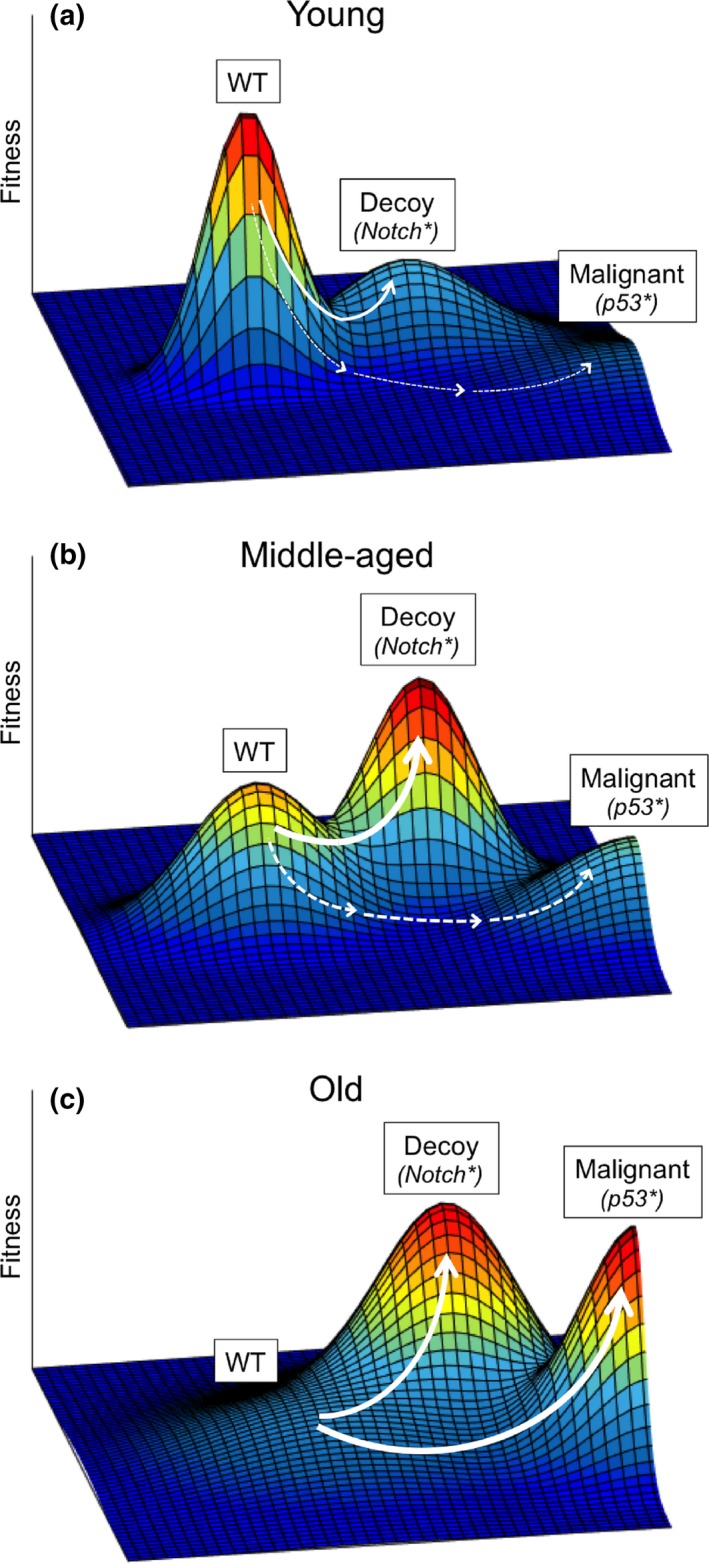
Proposed model for fitness landscape changes with age. (a) The young fitness landscape favors cell occupancy of the evolved adaptive peak (with “wild‐type,” WT, phenotype), but disfavors more malignant phenotypes. Some selection for potentially oncogenic mutations occurs, but with a bias toward “decoy peaks.” While progression up the decoy peak may be limited by the required passage through lower fitness intermediates, the small size of epithelial progenitor pools could facilitate such transitions through neutral drift. Alternatively, a single mutation, such as in Notch1, could mediate the “jump” to the other peak. (b) By middle age, fitness landscapes engender greater selection for the phenotypes that occupy decoy peaks (often with Notch1 mutations); while partially transformed, phenotypes of cells on these decoy peaks are more benign with reduced malignant potential. (c) At older ages, further tissue degradation and damage accumulation result in a landscape that increases the odds of mutational adaptation toward both benign (decoy) and more malignant phenotypes. Clones with more malignant phenotypes are more likely to progress toward cancer. Arrow thickness reflects hypothetical probabilistic phenotypic and fitness effects of mutation. The x‐y plane represents potential genetically and epigenetically encoded somatic cell phenotypes. Note that for simplicity, this model does not incorporate roles for age‐dependent mutation accumulation, which should clearly contribute to cancer risk with age. We also note that while experimental and observational data support changes in somatic selection in aging tissues, the shapes of these landscapes are hypothetical

For this model, we incorporate a previously substantiated concept—that the fitness impact of mutations can be highly context‐dependent. In particular, previous studies have demonstrated that oncogenic mutations are differentially selected for in tissues of young and old mice, with adaptation promoted in the aged context (Henry et al., 2015; Henry, Marusyk, Zaberezhnyy, Adane, & DeGregori, 2010; Parikh, Shuck, Gagea, Shen, & Donehower, 2018; Rozhok, Salstrom, & DeGregori, 2014; Vas, Wandhoff, Dörr, Niebel, & Geiger, 2012). Young tissues are inherently tumor suppressive, as mutations (including oncogenic ones) that change phenotype should typically result in loss of the mutated clone from the progenitor cell pool (DeGregori, 2013). Moreover, restoring a more youthful microenvironment, such as through blocking inflammation, can mitigate aging‐dependent oncogenic selection (Henry et al., 2015). Thus, consistent with the well‐established role of environmental factors in evolution (Eldredge, 1999), data support that the dramatic changes in fitness landscapes as we age impact clonal evolution in somatic tissues.

But what about support for “decoy fitness peaks”? There are numerous examples from the literature where more benign competition can disfavor somatic progression toward malignancy. In mice, transgenic expression of survivin, an inhibitor of apoptosis, in skin cells retards the expansion of clones with TP53 mutations by preventing the UV‐induced apoptosis of niche‐competing nontransformed cells (Zhang et al., 2005). In a mouse model of T‐cell Acute Lymphoblastic Leukemia (T‐ALL), bone marrow progenitors of varying relative fitness altered T‐ALL development, where the most fit, highly competitive progenitors completely suppressed T‐ALL development (Martins et al., 2014). In the context of ionizing radiation exposure of mice, the temporary inhibition of p53 in hematopoietic progenitors greatly improved their fitness, thus reducing radiation‐induced lymphomagenesis (Lee et al., 2015), consistent with earlier work demonstrating the ability of unirradiated wild‐type progenitors to suppress the expansion of irradiated p53‐mutant progenitors (Bondar & Medzhitov, 2010; Marusyk, Porter, Zaberezhnyy, & DeGregori, 2010). For the unavoidable context of aging, while aged hematopoietic competition enables development of Bcr‐Abl‐initiated leukemias, provision of young and thus fit hematopoietic competition can prevent leukemogenesis (Henry et al., 2010). Returning to humans, studies of clonal hematopoiesis of the elderly reveal that the spectrum of DNMT3A mutations driving clonal expansions in old age diverges from those associated with leukemias: Non‐R882 mutations make up the majority of mutant DNMT3A clonal hematopoiesis, but represent the minority of DNMT3A mutant acute myeloid leukemias (Atlas, 2013; Buscarlet et al., 2017; Genovese et al., 2014; Jaiswal et al., 2014; McKerrell et al., 2015; Xie et al., 2014), suggesting that the more dominant expansions present substantially less risk of leukemic progression than others. Similarly, BRAF mutations are much more common in lung premalignant lesions than in adenocarcinomas (Izumchenko et al., 2015), raising the possibility that BRAF mutations in lung epithelium could similarly reside on decoy adaptive peaks. In each case, it appears that the presence of more fit (at least relative to the damaged or aged pool) competition can limit oncogenesis or direct oncogenesis toward a less malignant path. Together with these prior results, the Martincorena et al and Yokoyama et al studies suggest that potentially oncogenic mutations (including epigenetic changes) can be adaptive without conferring a high risk of cancerous progression and that such decoy adaptive states could limit cancer development. We hypothesize that by providing an adaptation to the aged esophageal tissue environment, Notch1 mutations offer a less malignant alternative to a more oncogenic path, thus reducing cancer risk (at least until older ages). The relatively more benign Notch1 mutations not only reduce the chances that such cells accumulate additional oncogenic mutations (as they have already attained a higher position on the fitness landscape, leaving “less room for improvement”; see Figure 1), but can also serve as competitors for more malignant clones (as the Notch‐mutated clones are better adapted to the aged environment than the “wild‐type” cells).

It is important to remember that selection will drive progression toward a *local* fitness maximum, irrespective of whether the local fitness maximum impedes or enhances access to a global one. Progression up one adaptive peak obviates or reduces the fitness benefit of taking another. And once on a local but inferior fitness peak, progression to a higher peak becomes constrained by the requirement to progress via lower fitness intermediates (the valley between peaks in Figure 1). In this light, these studies suggest that while we cannot prevent aging (or the mutations that have occurred during life, whether due to intrinsic or extrinsic causes), we might be able to develop preventative and therapeutic strategies to favor more benign somatic evolutionary paths for cells (Maley, Reid, & Forrest, 2004). Given the risks and difficulties of genetically manipulating normal somatic cells, such an endeavor will likely involve interventions that engender tissue microenvironments that disfavor more malignant phenotypes. In fact, previous studies indicate that interventions can create tissue environments that disfavor more malignant phenotypes (Henry et al., 2015; Ibrahim‐Hashim et al., 2017; Mazzone et al., 2009), although it is not clear if these alterations are impacting decoy fitness states.

Of course, there are other potential explanations for the underrepresentation of Notch1 mutations in esophageal cancers. Perhaps key drivers accumulate earlier in life, when cells with Notch1 mutations constitute a small fraction of the epithelial progenitor pool; we might then assume that Notch1 mutations are no longer adaptive in clones with these drivers of malignancy (thus explaining their underrepresentation in cancers), while being adaptive in progenitors without such drivers. Alternatively, Notch1 mutant clones could represent dead‐end cellular expansions, which lose further self‐renewal activity (and are consequently less susceptible to further malignant progression)—“fitness sinks” rather than fitness peaks (akin to nevi in the skin). Notch1 mutant clones could even promote carcinogenesis noncell autonomously, as Yokoyama et al propose (Yokoyama et al., 2019). Nonetheless, while not the only possible interpretation, we posit that the concept of decoy fitness peaks provides an explanation consistent with available data. Clearly, this hypothesis will need to be tested, and with additional experimental evidence (such as through sequential sampling from the same individuals over decades, or by using animal and computational models) could be better supported, modified, or rejected. Still, if substantiated, understanding how decoy fitness peaks limit cancer development could enable researchers to design interventions that exploit decoy peaks (or create new ones) for cancer prevention and therapy.

Natural selection does not act to prevent mutation‐driven clonal expansions per se, but to prevent malignant growths from limiting reproductive success. We speculate that natural selection over millions of years has sculpted tissues and genetic networks to favor decoy adaptive peaks in what we now consider our middle ages (40s and 50s). While the strength of natural selection to limit cancer and other manifestations of animal senescence wanes at older ages (Rozhok & DeGregori, 2016), there is no cliff. For humans, inclusive fitness, such as through grandparenting, should have provided some selective pressure to disfavor cancer and tissue dysfunction even beyond the years of likely reproduction (Brown & Aktipis, 2015). But since survival to later years (60s and 70s) was rare in ancestral times, the benefits of investments into cancer suppressive strategies acting at these older ages would have been greatly reduced. Still, for middle age years, perhaps investment in tissue landscapes that favored somatic evolution up decoy peaks was a better investment or more feasible than the maintenance of tissue landscapes that would disfavor all somatic evolution. These decoy peaks could also simply reflect the nature of somatic evolution, with natural selection favoring animals whose somas exploited these somatic trajectories as tumor suppressive strategies. In toto, we can consider two phases of aging—the middle‐aged period (where historical odds of survival were not as low, and thus contributions to future generations could be realized) and a late‐aged period (where survival odds were minimal, and thus additional investments in disease avoidance strategies would have doubtfully paid off) (Figure 1). For the middle‐aged phase (and to some extent in youth), we hypothetically evolved tissues that favor (or allow) adaptation by Notch1 and other mutations, which may not only impede more malignant somatic evolution, but might mitigate some aging‐related tissue function decline. At later aging phases, other mutations such as in TP53 can become adaptive (but in progenitor cell pools shrunken by prior adaptive Notch1 mutations), contributing to esophageal cancers.

In all, the reports by Martincorena, Yokoyama and colleagues reveal how natural selection may have sculpted somatic programs to minimize cancer risk and thus maximize individual fitness. Additional studies, both from the clinic and from experimental and computational models, will be required to delineate the potential role, if any, of decoy fitness peaks in tissue maintenance and tumor suppression, and how these mechanisms evolved.

## CONFLICT OF INTEREST

None declared.
